# Paracrine Effects of Bone Marrow–Derived Endothelial Progenitor Cells: Cyclooxygenase-2/Prostacyclin Pathway in Pulmonary Arterial Hypertension

**DOI:** 10.1371/journal.pone.0079215

**Published:** 2013-11-18

**Authors:** Dong-Mei Jiang, Jie Han, Jun-Hui Zhu, Guo-Sheng Fu, Bin-Quan Zhou

**Affiliations:** 1 Department of Cardiology, Biomedical Research (Therapy) Center, Sir Run Run Shaw Hospital, College of Medicine, Zhejiang University, Hangzhou, Zhejiang Province, China; 2 Department of Cardiology, The First Affiliated Hospital, College of Medicine, Zhejiang University, Hangzhou, Zhejiang Province, China; Vanderbilt University Medical Center, United States of America

## Abstract

**Background:**

Endothelial dysfunction is the pathophysiological characteristic of pulmonary arterial hypertension (PAH). Some paracrine factors secreted by bone marrow–derived endothelial progenitor cells (BMEPCs) have the potential to strengthen endothelial integrity and function. This study investigated whether BMEPCs have the therapeutic potential to improve monocrotaline (MCT)-induced PAH via producing vasoprotective substances in a paracrine fashion.

**Methods and Results:**

Bone marrow-derived mononuclear cells were cultured for 7 days to yield BMEPCs. 24 hours or 3 weeks after exposure to BMEPCs in vitro or in vivo, the vascular reactivity, cyclooxygenase-2 (COX-2) expression, prostacyclin (PGI_2_) and cAMP release in isolated pulmonary arteries were examined respectively. Treatment with BMEPCs could improve the relaxation of pulmonary arteries in MCT-induced PAH and BMEPCs were grafted into the pulmonary bed. The COX-2/prostacyclin synthase (PGIS) and its progenies PGI_2_/cAMP were found to be significantly increased in BMEPCs treated pulmonary arteries, and this action was reversed by a selective COX-2 inhibitor, NS398. Moreover, the same effect was also observed in conditioned medium obtained from BMEPCs culture.

**Conclusions:**

Implantation of BMEPCs effectively ameliorates MCT-induced PAH. Factors secreted in a paracrine fashion from BMEPCs promote vasoprotection by increasing the release of PGI_2_ and level of cAMP.

## Introduction

**Figure 1:**
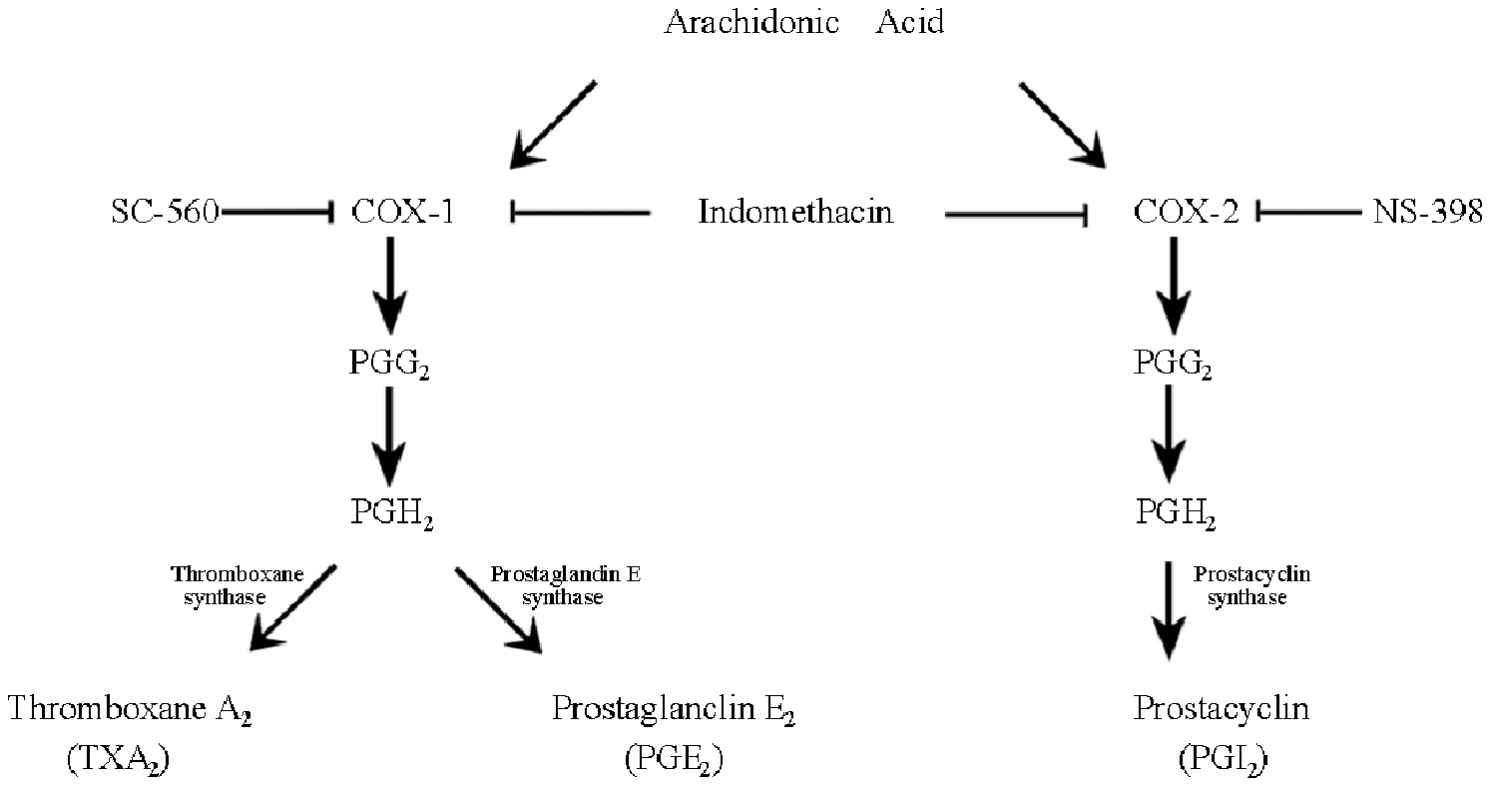
Metabolic pathways of arachidonic acid metabolism. Indomethacin: a nonselective COX inhibitor, NS-398: a selective COX-2 inhibitor, SC-560: a selective COX-1 inhibitor.

Pulmonary arterial hypertension (PAH) is a rare but progressive disease which is characterized by severe arteriopathy, including intimal hyperplasia, muscularization of the distal alveolar duct and pulmonary arteries, plexiform lesions and neointima which obstruct the pulmonary arteries and arterioles, and eventually leading to severe PAH, right heart failure and death within an average of 2–8 years from diagnosis [1], [2]. Dysfunction of endothelial homeostasis, e.g. imbalanced production between vasoconstrictors and vasodilators [3], is considered to play an important role in the initiation and development of PAH. The arachidonic acid pathway is suggested to play a central role in homeostasis of the endothelium and vascular smooth muscle cells as dysregulation of the cascade of arachidonic acid have been observed in patients and animal models with PAH. The cyclooxygenase enzymes (COX-1 and COX-2) catalyze the conversion of arachidonic acid to the intermediate prostaglandin H2, which is then converted to a series of prostanoids by cell-specific synthases (Fig. 1). Prostacyclin (PGI_2_) is the major metabolite of arachidonic acid, and an imbalance between PGI_2_ and thromboxane A_2_ (TXA_2_) has been demonstrated in patients with PAH [4]. Overexpression of PGI_2_ synthase in the lung protects against the development of hypoxia-induced PAH in mice and the administration of PGI_2_ analogues are currently in clinical treatment for PAH [5], [6], suggesting that PGI_2_ plays a key role as an endogenous regulator of endothelial homeostasis.

With the increased understanding of PAH pathogenesis, new therapeutic strategies are expected to lead to the improvement in the prognosis of PAH. Transplantation of endothelial progenitor cells (EPCs) has recently been proposed as one of the potential therapeutic strategies. In monocrotaline (MCT)-induced PAH rats, EPCs almost completely prevented the increase in right heart systolic pressure (RVSP) [7]. Furthermore, EPCs have been used to treat clinical PAH patients in our pilot study, showing some short-term efficacy [8].

Although there is increasing evidence showing the involvement of EPCs in neovascularization and vascular repair, the underlying mechanisms are poorly understood. It has been demonstrated that the early EPCs are able to release relevant growth factors and cytokines, such as vascular endothelial growth factor (VEGF), hepatocyte growth factor (HGF) and granulocyte colony-stimulating factor (G-CSF) [9]. These factors may augment the synthesis of vasoprotective substances like PGI_2_ in the vessel wall. However,the molecular networks regulating the accumulation and functions of COX-2/PGI_2_ are largely unknown.

Therefore, we first investigated whether bone marrow–derived endothelial progenitor cells (BMEPCs) could improve endothelial dysfunction in MCT-induced PAH and further provide evidence that BMEPCs may stimulate the production of PGI_2_ in pulmonary artery by a paracrine action.

## Materials and Methods

### Isolation and Culture of BMEPCs

Bone marrow (BM) was aspirated from the femora and tibiae of adult male syngeneic Fisher-344 rats, weighing 150–200 g. Mononuclear cells (MNCs) were isolated by density gradient (Ficoll-Paque, Amersham) centrifugation at 400×*g* for 30 minutes. The interphase layer of bone marrow-derived mononuclear cells (BMMNCs) were collected. The cells were washed twice with phosphate-buffered saline (PBS) before centrifugation at 400×*g* for 5 minutes. Then these cells were resuspended in Medium 199 supplemented with 20% fetal bovine serum (FBS), 50 U/mL penicillin, 50 µg/ml streptomycin, 2 mmol/L L-glutamine, 50 ng/mL VEGF, 5 ng/ml basic fibroblast growth factor (bFGF), plated on gelatin-coated tissue culture flasks and incubated at 37°C with 5% CO_2_ for 7 days to produce BMEPCs, as described previously [10]. Culture medium was changed every 48 hours. By day 7, for fluorescent staining, adherent cells were first incubated with 10 µg/mL Dil-acetylated LDL (Molecular Probes, Eugene, OR, USA) at 37°C for 2 hours and later fixed with 2% paraformaldehyde for 10 minutes, followed by incubation with FITC-labeled BS-1-Lectin (Sigma, St Louis MO, USA) at 37°C for 1 hour. The cells were identified under a fluorescence microscope and double-positive fluorescence was identified as differentiating BMEPCs. In some experiments, BMMNCs (day 0, 5×10^6^ cells) were seeded for 24 hours and used as controls.

### Preparation of Conditioned Medium and ELISA

On the 7th day of BMEPCs culture, adherent cells were collected and replated on 6 well culture dishes at a density of 5×10^6^ cells per well. 24 hours later, the original medium (1 mL/well) was changed with fresh Medium 199 (M199) without any supplement. Then after another 24 hours, the culture supernatant was centrifuged, sterile filtered with a 0.22 µm filter (Millipore, Billerica, MA, USA) and used as conditioned medium (CM). VEGF level was measured by enzyme-linked immunosorbent assay according to the instructions of the manufacturer (Raybiotech, Norcross GA, USA) and normalized to 5×10^6^ cells. BMMNCs (day 0,5×10^6^ cells) were cultured with fresh M199 of the same volume with no supplement for 24 hours and served as a control.

### Gene Transduction

Replication-deficient recombinant adenoviral vectors encoding green fluorescent protein (Ad-GFP) under the control of a cytomegalovirus promoter were generated according to the method previously described [11]. When the cells reached 70–80% confluence, 50 µL Ad-GFP (5×10^9^ plaque-forming units, pfu) was added into the culture medium and co-cultured with BMEPCs for infection at the most appropriate multiplicity of infection (MOI) = 50 for 72 hours [12]. Cells were washed free of virus and studied for GFP expression efficiency, using confocal fluorescent microscope. In separate experiments, Ad-GFP labeled BMEPCs (5×10^6^) suspended in 1 mL saline were implanted into the pulmonary circulation of rats at 21 days after MCT injection via the external jugular vein. 7 days later, the lungs and pulmonary arteries were collected and embedded in optimum cutting temperature compound (OCT) (Tissue-Tek) (Sakura Finetechnical Co) at −20°C. Then flash-frozen sections were examined using a laser confocal microscope (LSM 510; Zeiss, Oberkochen, Germany).

### Animal Models and Experimental Protocol

MCT (Sigma, St. Louis, MO) was dissolved in 1 N HCl, neutralized with 1 N NaOH, diluted with saline. Male syngeneic Fisher-344 rats (200–250 g, Laboratory Animal Center, Chinese Academy of Science, Shanghai) were housed with a 12/12 hours light/dark cycle and given an unrestricted food and water supply. In the invitro experiment, on day 1, animals received a single intraperitoneal injection of MCT (60 mg/kg body weight, BW) to induce PAH or saline to serve as a control. On day 21, RVSP was recorded to confirm the presence of PAH, then the rats were sacrificed and the pulmonary arteries were harvested to study vascular activity, protein expression and the cAMP content. In the invivo experiment, rats were injected with saline or MCT. 21 days later, MCT-treated rats were randomized to receive M199 (MCT alone), (5×10^6^) BMEPCs, or BMEPCs-CM. 21 days later (42 days after MCT), hemodynamic parameters were recorded, then the rats were euthanized, and lungs, hearts and pulmonary arteries were collected for analysis as described in the invitro experiment. All of the experimental procedures in this study were approved by the Institutional Animal Care and Use Committee of Zhejiang University Medical Center and conformed to the National Institutes of Health Guide for the Care and Use of Laboratory Animals (NIH Pub. No. 85-23, Revised 1996).

### Hemodynamic Parameters

21 days after MCT injection, the rats were anesthetized with intraperitoneal injections of 4% chloral hydrate (10 mL/kg, BW) and intubated, mechanically ventilated with a small-animal ventilator (ALC-V8, ALC Inc. Shanghai). The right cervical area was shaved and cleaned with 70% ethanol, and the external jugular vein was catheterized with a 3F microtip catheter (Biopac System, Goleta, CA, USA) into the RV to measure RVSP. Then rats were allowed to recover. 21 days later (42 days after MCT), hemodynamic measurements were recorded again. Then the heart was excised and the ratio of right to left ventricular plus septal weight (RV/LV+IVS) was determined as Fulton’s index. The lungs were weighed and calculated the ratio of L/BW.

### Morphometric Analysis

Formalin-fixed rat lungs were paraffin-embedded and the sections (6 µm) were stained with hematoxylin-eosin according to routine histopathologic procedures. Light microscopic slides were analyzed by two external observers in a blinded fashion. Images were captured with a microscope digital camera and analyzed with an image analysis program (NIH Image). 15 vessels (<100 µm in diameter) of each rat were counted. The medial wall thickness was calculated with the formula: [(external diameter−internal diameter)/external diameter]×100 [13].

### Vascular Reactivity Analysis

In the invitro experiment, the pulmonary arteries were rapidly separated and placed in cold oxygenated Krebs’ solution composed of (mmol/L): NaCl 118.3, KCl 4.7, CaCl2 2.5, KH2PO4 1.2, MgSO4-7H2O 1.2, NaHCO3 25 and glucose 11.1. The right and left pulmonary arteries were carefully dissected free of fat and connective tissue, and cut into 3 mm wide rings. Then under sterile tissue culture conditions, vascular rings were exposed to BMMNCs and BMEPCs (5×10^6^ cells) in 1 well (3 mL volume) of a 6-well tissue culture plate for 24 hours at 37°C in humidified atmosphere of 5% CO_2_. After an overnight incubation, the vessel rings were mounted in an organ bath filled with 5 mL of Krebs’ solution at a temperature of 37°C and continuously bubbled with 95% O_2_/5% CO_2_. The rings were attached to a force-displacement transducer connected to an amplifier to record tension changes (Meiyi Science Inc. Nanjing). Tissues were allowed to equilibrate under 1 g resting tension for 60 minutes, during which time the bath solution was replaced every 15 minutes and the resting tension was readjusted when necessary. After equilibration, a reference contraction to 60 mmol/L KCl was obtained and then rings were washed until tension returned to the baseline. The procedure was repeated twice. Vascular rings were contracted with increasing phenylephrine (PHE, 10^−9^ to 10^−5^ mol/L), then a cumulative concentration-response curve was obtained. To measure vasorelaxation, rings were first preconstricted with PHE (10^−5^ mol/L), after reaching a steady-state contraction, increasing concentration of acetylcholine (ACH, 10^−9^ to 10^−5^ mol/L) and sodium introprusside (SNP, 10^−9^ to 10^−5^ mol/L) were added, and the percentage of relaxation of the PHE contraction was measured. In some experiments, concentration-response curves were obtained in rings pretreated with the COX-2 inhibitor, NS-398 (10^−5^ mol/L) for 30 minutes.

### Western Blot Analysis

Total protein from pulmonary arteries were mechanically homogenized in ice-cold lysis buffer. Equal amounts of protein were separated and transferred, then the membranes were separately incubated overnight with the following primary antibodies: mouse monoclonal antibodies against COX-1, COX-2 (1∶200 dilution, Santa Cruz, Biotech), rabbit polyclonal antibodies against inducible NO synthase (iNOS, 1∶200 dilution, Santa Cruz, Biotech), endothelial NO synthase (eNOS, 1∶200 dilution, Santa Cruz, Biotech), rabbit polyclonal antibody against PGI_2_ synthase (PGIS, 1∶250 dilution, Abcam, Cambridge, UK). Then blots were incubated with horseradish peroxidase-conjugated secondary antibodies. Quantification of the bands was carried out using densitometric analysis software (Quantity One, Bio-Rad, CA, USA).

### Immunohistochemical Analysis

21 days after BMEPCs implantation, pulmonary arteries were embedded in paraffin and cut into 5 µm sections. After an overnight incubation with polyclonal anti-COX-2 antibody (1∶50 dilution, Santa Cruz) at 4°C, the sections were incubated with the secondary antibody and counterstained with hematoxylin-eosin. Negative controls were performed in absence of antibody. The staining was examined using light microscope and analyzed with a computer-assisted color image analysis system (Image-Pro Plus 6.0).

### Measurements of 6-keto-Prostaglandin F_1α_, Prostaglandin E_2_ and Thromboxane B_2_

Pulmonary arteries isolated from rats, that were injected with M199, BMEPCs or BMEPCs-CM, were incubated in Krebs’ solution in a CO_2_ incubator at 37°C for 30 minutes. After incubation, the Krebs’ solution was collected and the concentrations of 6-keto-Prostaglandin F_1α_ (6-keto-PGF_1α_), Prostaglandin E_2_ (PGE_2_) and Thromboxane B_2_ (TXB_2_) were measured using their respective enzyme immunoassay kits (Enzo Life Sciences) according to the manufacturers’ instructions. In some experiments, pulmonary arteries were incubated with indomethacin (10^−5^ mol/L), NS-398 (10^−5^ mol/L), or the COX-1 inhibitor SC-560 (10^−6^ mol/L) for 30 minutes before measurement of 6-keto-PGF1α.

### Measurement of cAMP

Pulmonary arteries were soaked in 1 mL of 0.5 mmol/L acetic acid, then boiled and centrifuged. The supernatants were preserved for measurement by cAMP radioimmunoassay kit (Zhongsheng Beikong bio-technology and science Inc. Beijing, China). In some experiments, pulmonary arteries were incubated with indomethacin (10^−5^ mol/L), SC-560 (10^−6^ mol/L), or NS-398 (10^−5^ mol/L) for 30 minutes before measurement.

## Statistical Analysis

The data were expressed as means ± S.E.M. Differences among different groups were assessed by one-way ANOVA and the difference between means was further evaluated with the least significant difference test (LSD) if a statistical difference was detected in the ANOVA analysis. SPSS 16.0 was used for the statistical analysis and P<0.05 was considered significant.

## Results

### Characteristics of BMEPCs

**Figure 2:**
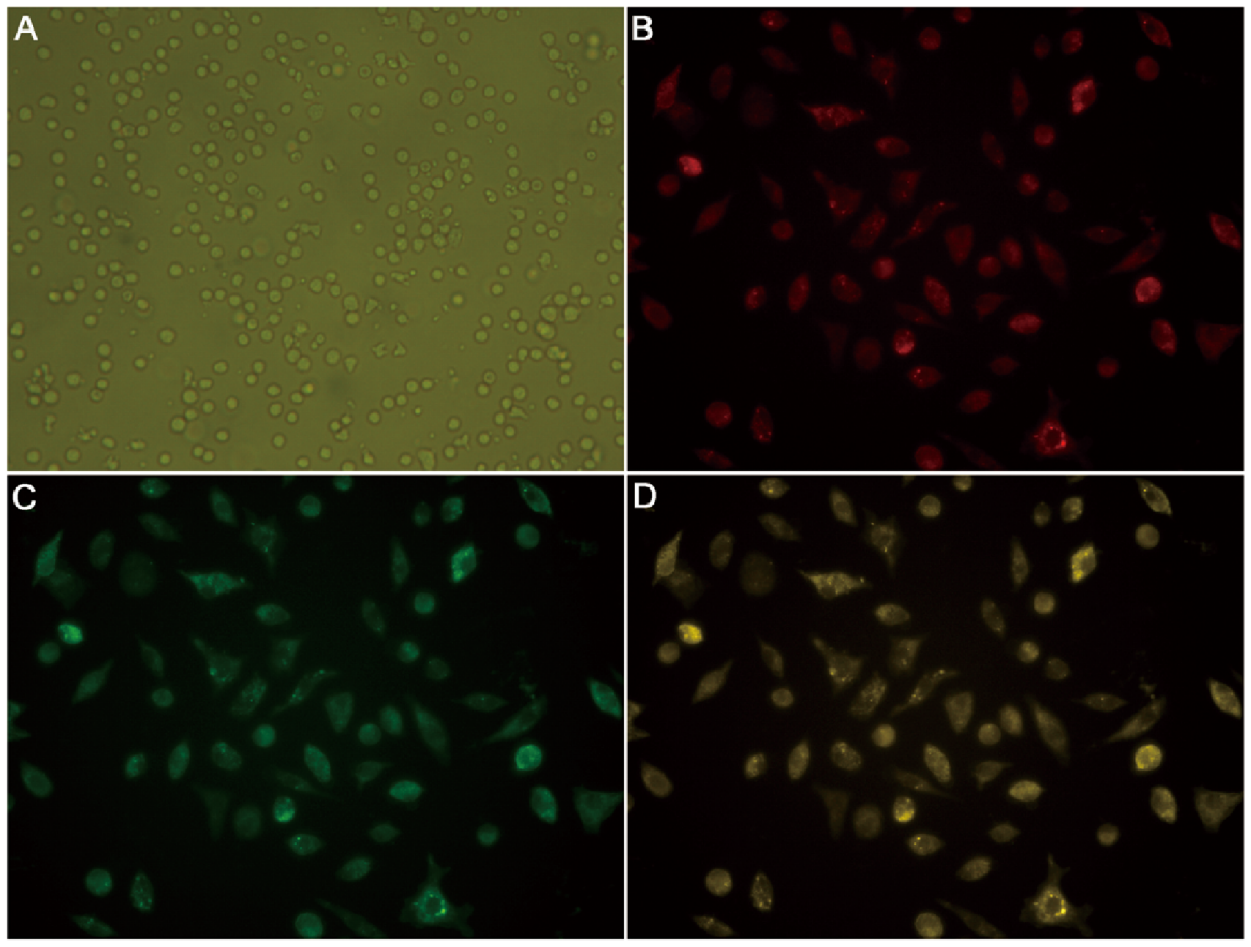
Characterization of BMEPCs derived from rat bone marrow. (A) BMMNCs (0 day) were globe-like shape (×200). (B) After 7 days, Dil-ac-LDL positive cells were red. (C) FITC-Lectin-BS-1 positive cells were green. (D) Dil-ac-LDL/FITC-Lectin-BS-1 double-positive cells were differentiating BMEPCs (×400).

BMMNCs were round and varied in size after isolation (Fig. 2A). After 7 days of culture, BMMNCs exhibited spindle-shaped. Adherent cells were able to intake Dil-ac-LDL, showing red color under the confocal microscope (Fig. 2B), and also capable of binding with FITC-Lectin-BS-1, making them green in color (Fig. 2C). Double-positive cells were dividing BMEPCs (Fig. 2D).

### VEGF and Distribution of BMEPCs

**Figure 3:**
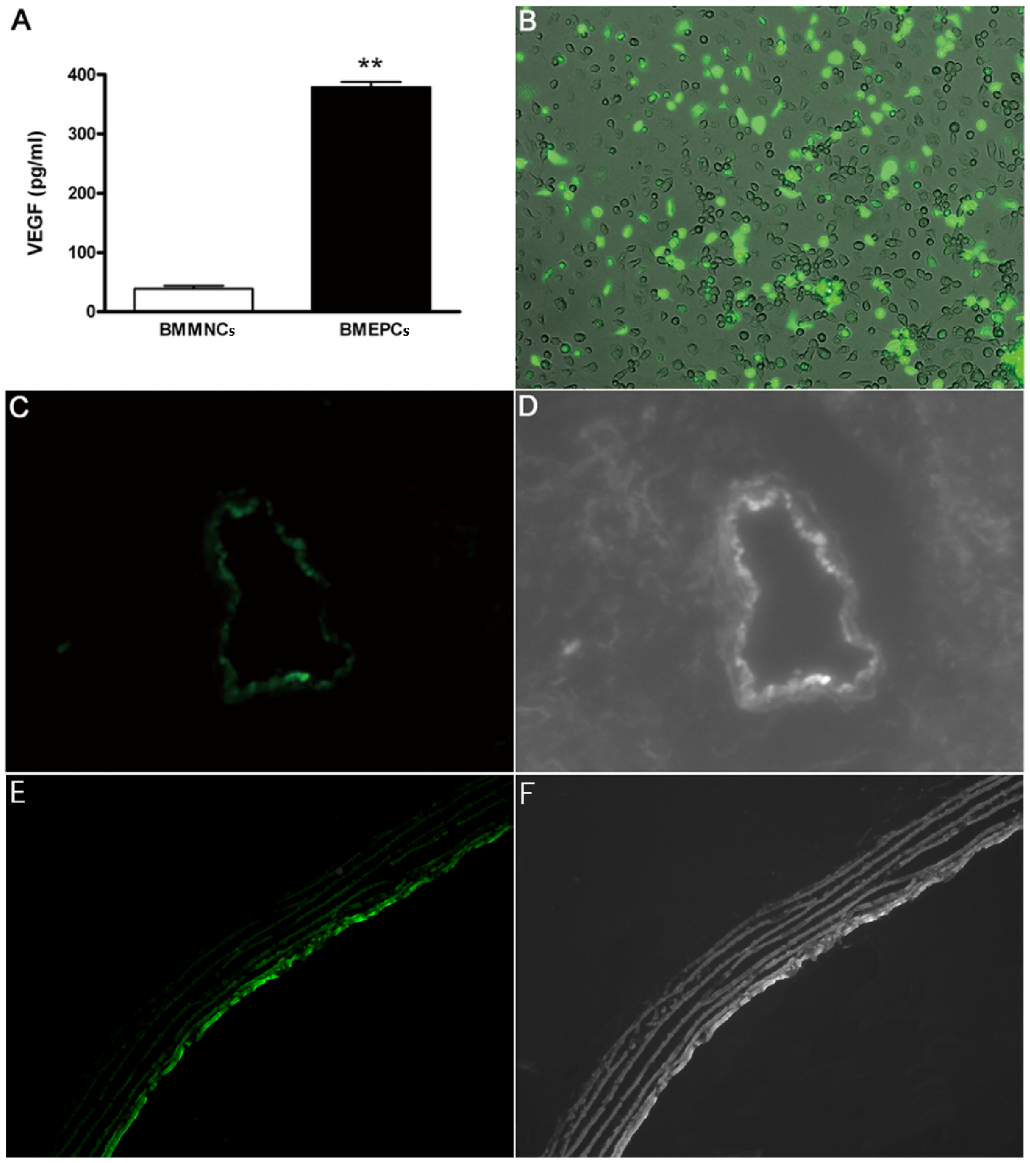
The expression of VEGF and distribution of Ad-GFP labeled BMEPCs. (A) Bar diagram representing significantly elevated content of VEGF in conditioned medium from BMEPCs (**P<0.01, n = 6). (B) Green fluorescent expression of adenovirus-green fluorescent protein (Ad-GFP) on BMEPCs (×100). (C, D) Implanted Ad-GFP labeled BMEPCs were distributed into lung tissues. (E, F) Ad-GFP labeled BMEPCs were incorporated into pulmonary arterioles (×200).

The secretion pattern of growth factors and cytokines in the BMEPCs-CM was investigated as previously reported [14]. Among all the factors detected, VEGF was proved to be the major factor. We also found the level of VEGF was significantly higher in BMEPCs-CM when compared with BMMNCs-CM (Fig. 3A). To investigate the distribution of BMEPCs, we used recombinant adenovirus carrying GFP to transfect BMEPCs (Fig. 3B). Then the GFP labeled BMEPCs were injected from internal jugular vein into pulmonary circulation on day 21 after MCT-induced PAH. 7 days later, the cells were found to be distributed into lung tissues (Fig. 3C, 3D) and engrafted into pulmonary arteries of MCT-treated rats (Fig. 3E, 3F). Our findings were consistent with previous ones [10].

### Effects of BMEPCs on Pulmonary Arteries in vitro

**Figure 4:**
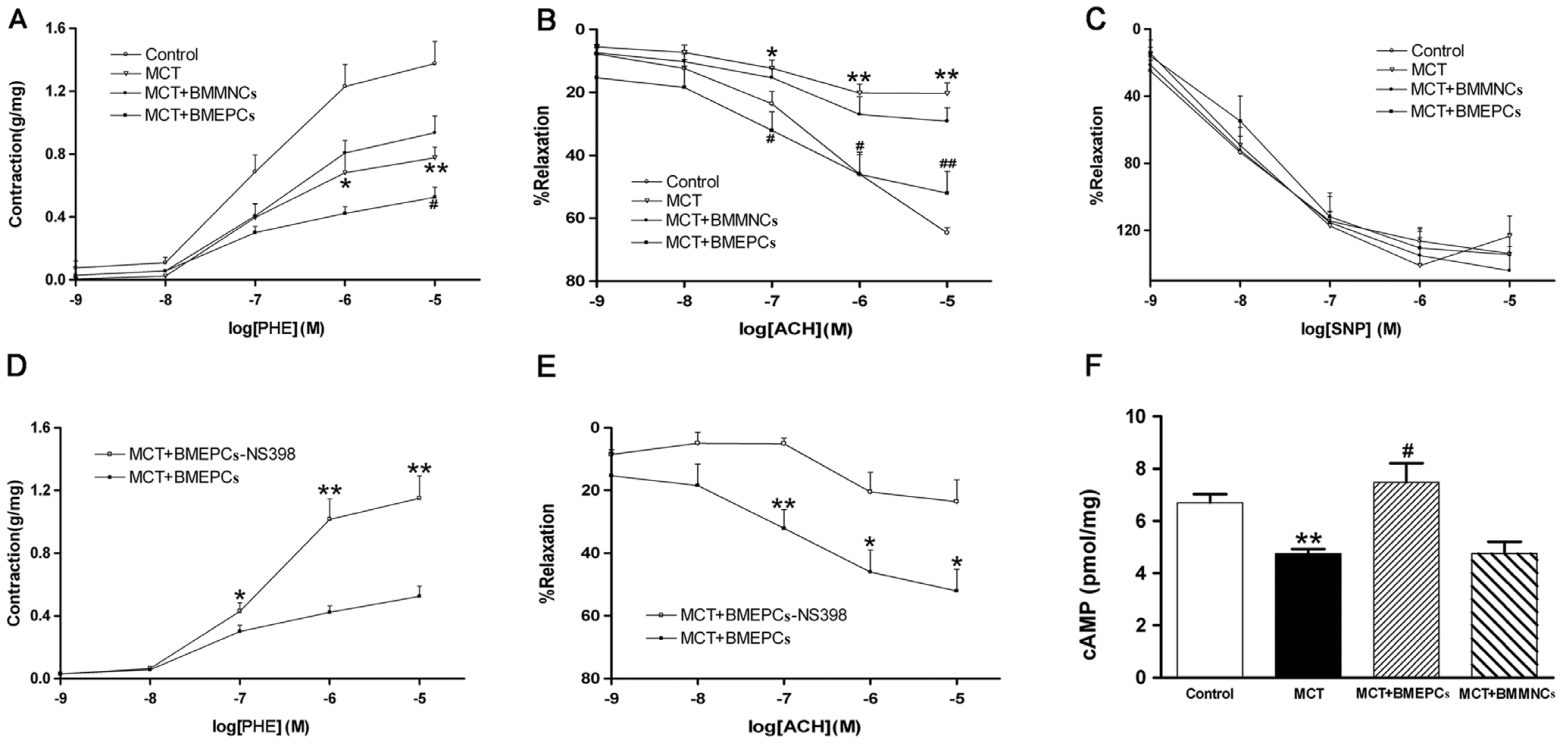
The effects of BMEPCs on pulmonary vascular reactivity. (A) PHE-induced contraction in pulmonary arteries. (B, C) ACH and SNP-induced relaxation. (D, E) The effect of NS-398 on PHE-induced contraction and ACH-induced relaxation. The contractile response was measured and presented in grams per milligram tissue weight for pulmonary arteries. Relaxation was expressed as the percentage of precontraction with PHE. (F) Bar diagram representing reduced content of cAMP in MCT group, but significantly enhanced by BMEPCs. While BMMNCs did not improve the cAMP level. (*P<0.05, **P<0.01 vs. control; ^#^ *P<*0.05, ^##^ *P<*0.01 vs. MCT group, n = 8).

**Figure 5:**
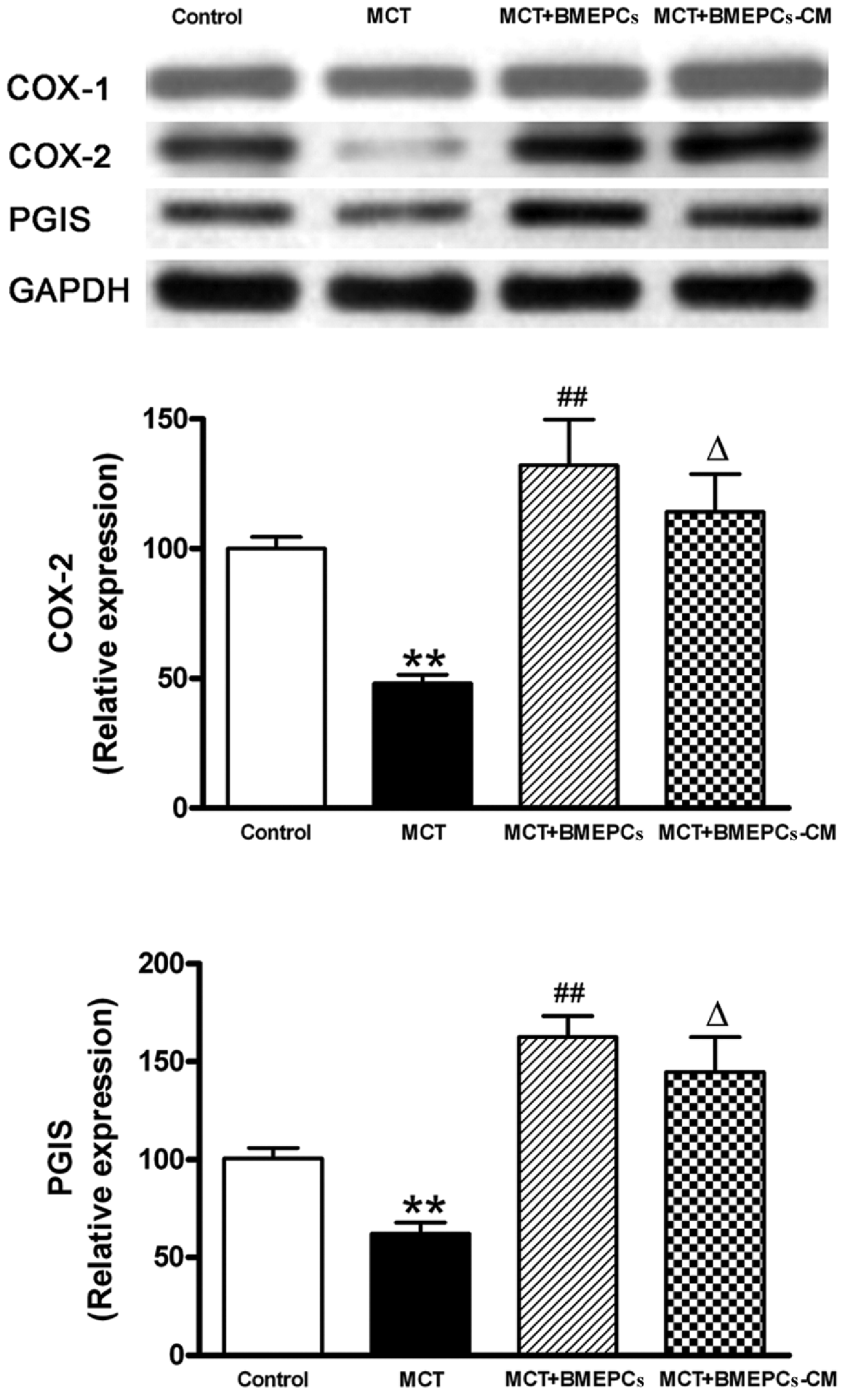
COX-2, PGIS and COX-1 expression in pulmonary arteries after exposure to BMEPCs and BMEPCs-CM quantified by western blot analysis. (**P<0.01 vs. control; ^Δ^ *P<*0.05,^ ##^ *P<*0.01 vs. MCT group, n = 6).

Previous studies have demonstrated that BMEPCs could repair the injured relaxation of pulmonary arteries in hypoxia induced PAH. To address the effects of BMEPCs on pulmonary arteries in MCT induced PAH, we analyzed the vascular reactivity in vitro. The PHE-induced contraction was reduced in MCT group as compared with control group, and the contraction in BMEPCs group was further reduced as compared with MCT group, while, the PHE response was not significantly different between BMMNCs group and MCT group (Fig. 4A). The ACH-induced relaxation was significantly reduced in MCT group as compared with control group (20.37±3.29% vs. 64.69±1.61%), but was significantly potentiated in BMEPCs group (54.79±5.56% vs. 20.37±3.29%), whereas the maximal relaxation was not different between BMMNCs and MCT groups (Fig. 4B). In addition, the concentration-dependent relaxation to exogenous NO donor, SNP, was not significantly different in all experimental groups (Fig. 4C), suggesting reduced production of endothelium-derived vasodilators in experimental PAH. Pretreatment with a selective COX-2 inhibitor, NS-398, increased PHE-induced contraction (Fig. 4D), but reduced ACH-induced relaxation in arteries treated with BMEPCs (Fig. 4E), thereby suggesting that up-regulation of COX-2 was responsible for the improved effect of BMEPCs on PAH relaxation. After exposure to BMMNCs and BMEPCs for 24 hours, the pulmonary arterial content of cAMP was significantly increased in BMEPCs group compared with MCT group (Fig. 4F). And western blot analysis on pulmonary arteries showed COX-2 and PGIS protein expression were higher in BMEPCs and BMEPCs-CM groups than in MCT group (Fig. 5).

### Effects of BMEPCs Implantation on PAH in vivo

**Figure 6:**
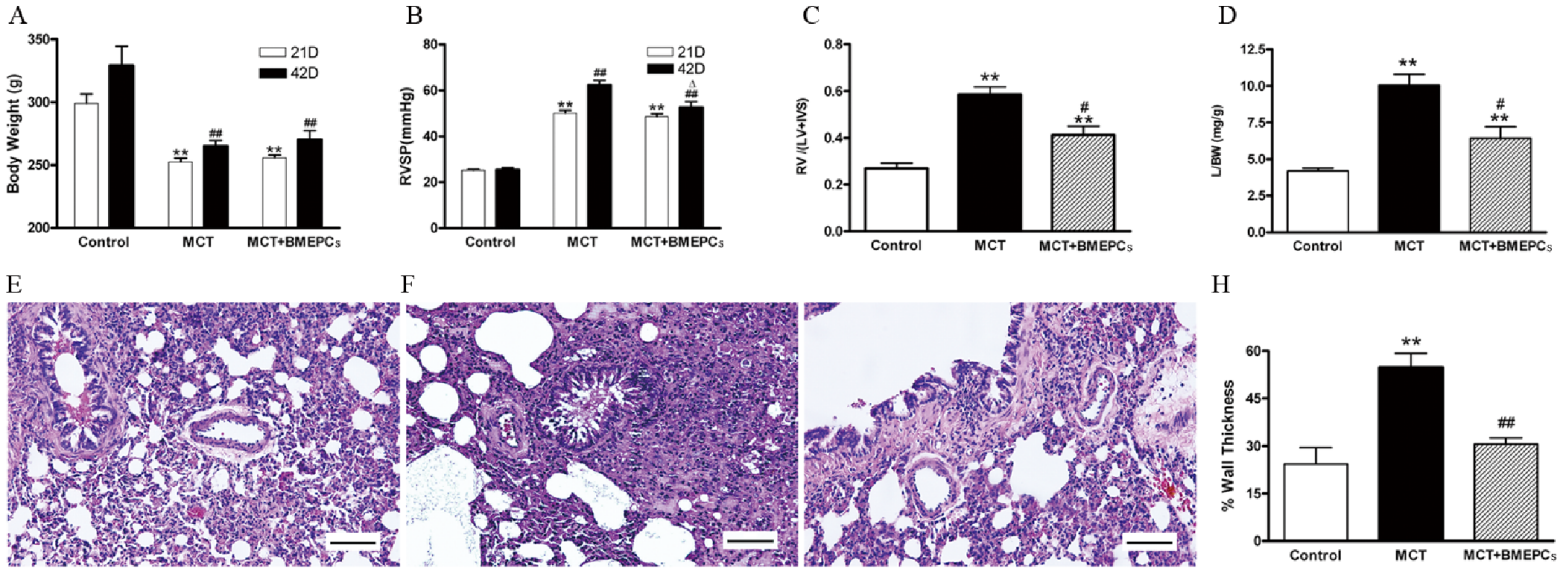
The effects of BMEPCs implantation on PAH in vivo. (A) The body weight change. (**P<0.01 vs. 21 days control; ^##^ *P<*0.01 vs. 42 days control, n = 6). (B) The change of right heart systolic pressure (RVSP) (**P<0.01 vs. 21 days control; ^##^ *P<*0.01 vs. 42 days control; ^Δ^ *P<*0.05 vs. 42 days MCT group, n = 6). (C) The ratio of right to left ventricular plus septal weight [RV/(LV+IVS)] on day 42 after MCT injection (**P<0.01 vs. control; ^#^ *P<*0.05 vs. MCT group, n = 6). (D) The ratio of lungs to body weight (L/BW) (**P<0.01 vs. control; ^#^ *P<*0.05 vs. MCT group, n = 6). Representative hematoxylin-eosin staining of paraffin embedded rat lung tissue. (E) Pulmonary arterioles of the control group showing very thin media. (F) Markedly thicken pulmonary arterioles walls on day 42 after MCT injection. (G) Pulmonary arterioles treatment with BMEPCs. (H) The percentage of wall thickness (15 vessels per rat). Total magnification, ×400. Scale bar = 50 µm. (**P<0.01 vs. control; ^##^ *P<*0.01 vs. MCT group, n = 6).

After finding that BMEPCs could improve endothelial dysfunction in MCT-induced PAH in vitro, we then went on to investigate the effects of BMEPCs implantation on PAH in vivo. On the 21st day after MCT injection, rats treated with MCT became weak, lethargic and showed a dull coat. On the 42nd day after MCT injection, body weight was lower in MCT group than control group (265.17±4.48 vs. 329.33±15.18). This decrease was not significantly prevented by BMEPCs implantation (Fig. 6A). In comparison with control rats (25.42±0.95 mmHg), MCT-treated rats had a significant increase in RVSP on day 21 (50.12±1.22 mmHg), with a further increase on day 42 (62.37±1.98 mmHg). However, the delivery of BMEPCs prevented the further increase in RVSP at day 21 (52.66±2.41 mmHg) (Fig. 6B). Moreover, BMEPCs implantation attenuated the increase in RV/LV+IVS ratio compared to MCT group (0.41±0.04 vs 0.59±0.03) (Fig. 6C). Concomitantly, the L/BW ratio also decreased from10.07±0.76 to 6.43±0.80 (Fig. 6D). Histological examination revealed that, in comparison with control group (Fig. 6E), a single injection of MCT resulted in severe hypertrophy of pulmonary vessels (Fig. 6F). However, BMEPCs treatment markedly attenuated this change (Fig. 6G), and quantitative morphometric analysis also demonstrated a significant reduction of wall thickness in BMEPCs group (Fig. 6H).

**Figure 7:**
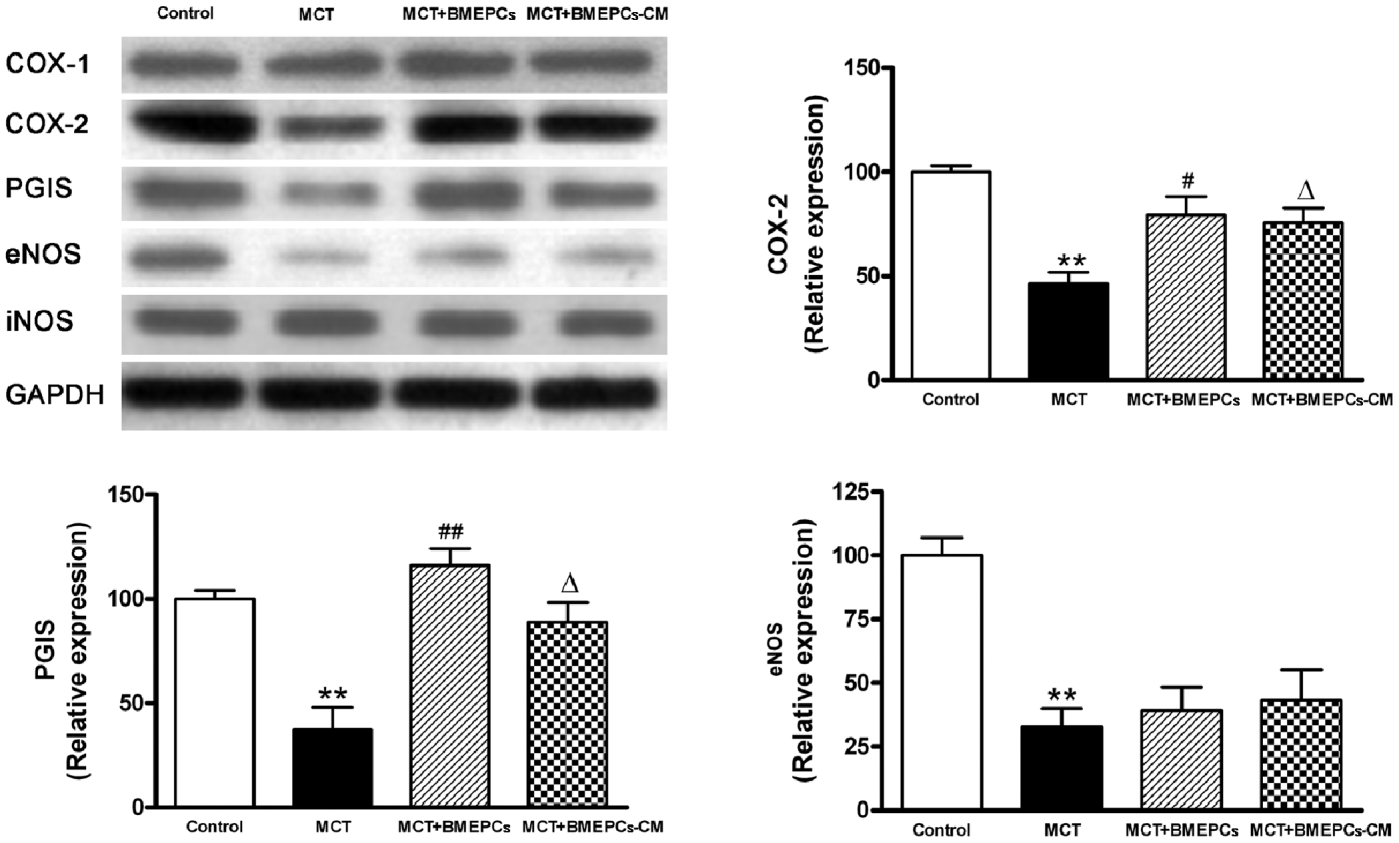
COX-2-PGIS-COX-1, eNOS and iNOS expression in pulmonary arteries after implantation of BMEPCs and BMEPCs-CM quantified by western blot analysis. (**P<0.01 vs. control; ^#^ *P<*0.05, ^Δ^ *P<*0.05, ^##^ *P<*0.01 vs. MCT group, n = 6).

Western blot showed that implantation of BMEPCs and BMEPCs-CM in vivo led to a significant up-regulation of COX-2 and PGIS, but not eNOS and iNOS in pulmonary arteries compared with MCT group. There was a down-regulation of eNOS expression in comparison with control group, but no change of iNOS in all groups (Fig. 7).

**Figure 8:**
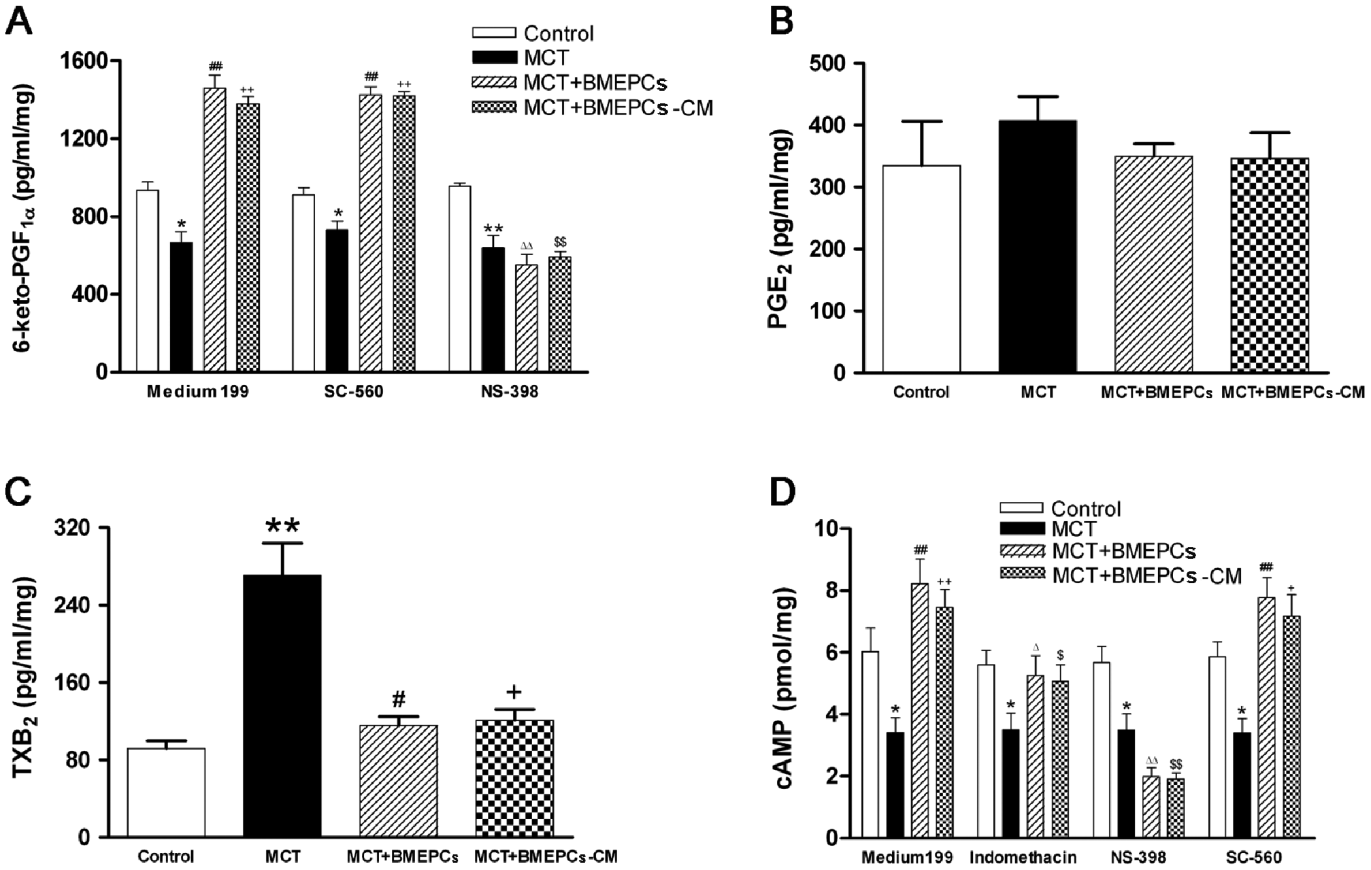
The effects of BMEPCs on the release of vasofactors. (A) The production of 6-keto-Prostaglandin F1α (6-keto-PGF1α) in pulmonary arteries. (B) The production of Prostaglandin E_2_ (PGE_2_). (C) The production of Thromboxane B_2_ (TXB_2_). (D) The content of cAMP in pulmonary arteries (*P<0.05, **P<0.01 vs. control; ^#^ *P<*0.05, ^+^ *P<*0.05 ^##^ *P<*0.01, ^++^ *P<*0.01 vs. MCT group; ^Δ^ *P<*0.05, ^ΔΔ^ *P<*0.01 vs. MCT+BMEPCs group incubation with Medium 199;^ $^ *P<*0.05, ^$$^ *P<*0.01 vs. MCT+BMEPCs-CM group incubation with Medium 199, n = 6).

After 30 minutes incubation of pulmonary arteries, the Krebs’ solution was collected and used to determine the release of vasofactors. The results showed that the release of 6-keto-PGF_1α_ was significantly reduced in MCT group, but increased in BMEPCs and BMEPCs-CM groups. Moreover, this change was sensitive to the COX-2 inhibitor, NS-398, but not the COX-1 inhibitor, SC-560 (Fig. 8A). The release of PGE_2_ was not affected in all groups (Fig. 8B), whereas, the level of TXB_2_ was significantly increased in MCT group compared with control group, but decreased in BMEPCs and BMEPCs-CM groups (Fig. 8C). The content of cAMP was also significantly increased in BMEPCs and BMEPCs-CM groups. The presence of a nonselective COX inhibitor, indomethacin or a selective COX-2 inhibitor, NS-398, could inhibit the increase of cAMP, whereas no similar effect was observed when treated with a selective COX-1 inhibitor, SC-560 (Fig. 8D).

**Figure 9:**
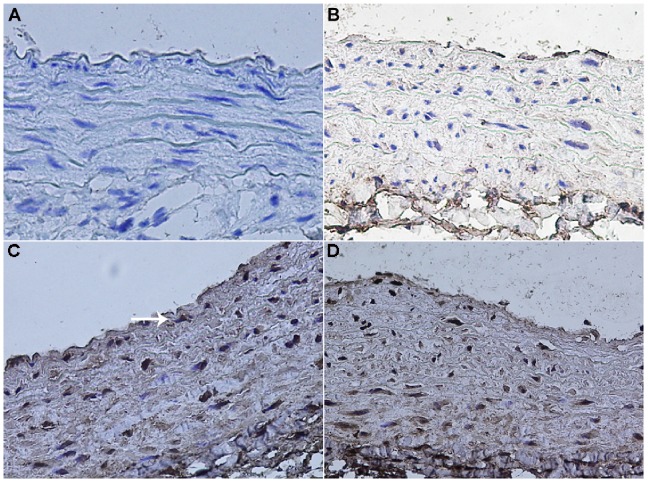
Immunohistochemical staining in pulmonary arteries of rats (arrow). (A) The negative control group. (B) The reduced expression of COX-2 in MCT group. (C, D) The increased expression of COX-2 in BMEPCs and BMEPCs-CM groups (×400).

Immunohistochemical experiments demonstrated that COX-2 was predominantly expressed in BMEPCs and BMEPCs-CM treated pulmonary arteries, and was localized in all three layers of vascular wall (Fig. 9).

## Discussion

In this study, we present the evidence that implantation of BMEPCs has the potential to attenuate PAH in MCT-treated rats. Furthermore, the observed effects appear to be partially mediated by activation of arachidonic acid metabolism via the COX-2/PGIS pathway.

Endothelial dysfunction is a hallmark of PAH and affects the production of vasoconstrictors and vasodilators, shifting the balance in favor of vasoconstrictors and ultimately resulting in pulmonary vascular remodeling [3]. Therefore, the recovery of endothelial function is a common target of treatments. It has been suggested that EPCs are important in maintaining vascular homeostasis, contributing to neovascularization and tissue recovery after ischemia and endothelial injury. There is now compelling evidence that transplantation of EPCs, including circulating EPCs, BMEPCs or gene modified-EPCs, can attenuate PAH in animal models or even in PAH patients [7], [8]. These studies contribute the benefit of EPCs to neovasculariztion and potential to differentiate into endothelial cells. However, neovasculariztion is sufficient in pulmonary circulation of PAH patients, and there are even plexiform lesions and neointima formation in some patients. Therefore, neovascularization of EPCs can not fully account for the protective effect on PAH. Another ability of EPCs to secrete paracrine factors is also worth further investigation.

Many prior studies have demonstrated that the transplantation of EPCs inhibits the apoptosis of endothelial cells, or prevents oxidative stress of PAH, partially through paracrine mechanisms [14]–[16]. The results of our study also provide evidence in support of this concept and demonstrate that the effect of BMEPCs may be dependent on the release of BMEPCs-derived vasoactive substances. It has been reported that cytokines could be released from BMEPCs, most probable are VEGF, transforming growth factor-β (TGF-β) and Interleukin-8 (IL-8) [14]. In our study, we found the release of VEGF was more prominent in the BMEPCs-CM group. It has been demonstrated that VEGF promotes angiogenesis by inducing the proliferation, differentiation, and chemotaxis of endothelial cells [17]. The administration of pulmonary artery-derived SMC to rats with MCT-induced PAH had therapeutic effects only when transduced in vitro with VEGF gene [18]. Likewise, the intravenous administration of fibroblasts transfected with VEGF gene was effective in reversing PAH [19]. It has also been demonstrated that VEGF induced COX-2 can lead to PGI_2_ formation, the main prostaglandin generated by endothelial cells [20]. Moreover, COX-2 plays an important role in VEGF-induced angiogenesis via p38 and JNK kinase activation pathways [21]. Therefore, we proposed that EPCs might stimulate COX-2/PGI_2_ formation in a paracrine fashion.

We first used an invitro approach to examine the effect of BMEPCs on isolated pulmonary arteries and explored its possible mechanism. The PHE or KCl-induced contraction and ACH-induced relaxation became impaired in the pulmonary arteries of MCT-treated rats compared with controls ones. These results were consistent with previous reports and they provided a plausible explanation that the structural changes of pulmonary arteries reduced responsiveness to vasoconstrictors and vasodilators [22], [23]. Whereas, our results showed endothelial-independent relaxation induced by SNP was not different in the groups studied. BMEPCs increased ACH-induced relaxation in pulmonary arteries, however, it was inhibited by the COX-2 inhibitor, NS-398. Therefore, BMEPCs may improve endothelial-dependent vasorelaxation in pulmonary arteries of MCT-treated rats through COX-2/PGI_2_ pathway. In contrast, incubation with mononuclear cells did not change the response to PHE and ACH in pulmonary arteries of MCT-treated rats. These findings demonstrated the selective effect of BMEPCs on pulmonary arteries. Further study showed that BMEPCs increased protein expression of COX-2/PGIS and content of cAMP. Therefore, improvement of relaxation is partly caused by high content of PGI_2_/cAMP in pulmonary arterial wall [24].

In the invivo experiment, we used a well-established model of PAH in rats induced by MCT [25], which injured endothelial cells of arteries and capillaries in the lungs. We found that intravenous administration of BMEPCs led to an improvement in pulmonary hemodynamic parameters and pulmonary remodeling. Ad-GFP labeled BMEPCs were incorporated into pulmonary beds in MCT-treated rats. These findings are consistent with previous reports that intravenously administered EPCs or vasodilator gene-transduced EPCs significantly attenuated the PAH in MCT-treated rats [4], [26], and these transplanted EPCs had been shown to be attracted to pulmonary arterioles and capillaries in MCT-treated rats and differentiated into mature endothelia [10], [26]. So far, there is disagreement on whether eNOS expression was reduced in PAH. In MCT-induced PAH rats, lung eNOS mRNA expression was reduced [27], however, in hypoxia-induced PAH rats, lung eNOS mRNA and protein levels were increased, but eNOS activity was reduced [28]. In addition, eNOS mRNA or protein expression was also reported to be increased in pulmonary vessels prepared from hypoxia-and MCT-induced PAH rats [29], [30]. The iNOS mRNA and protein expression were reported to be up-regulated in lung and small intrapulmonary vessels from chronic hypoxic rats [31]. In our study, the protein expression of eNOS was decreased in pulmonary arteries of MCT-treated rats, but no change was found in the protein expression of iNOS. The reasons for these inconsistent findings are unclear, but may reflect, at least in part, differences in the methods and animal models. In the invivo experiment, there are many complex mechanisms for regulating eNOS/iNOS expression, such as hemodynamic change and varying levels of active substrates. Therefore, detailed investigations will be needed to reveal these differences in each model. We also found that intravenous delivery of BMEPCs did not affect the protein expression of eNOS and iNOS, suggesting that eNOS/iNOS/NO pathway was probably not responsible for the observed effect of BMEPCs in this study. COX-1 was constitutively expressed and showed a similar expression level in all studygroups, whereas COX-2 and downstream PGIS expression were decreased in MCT-treated rats. Consistent with the invitro findings, BMEPCs only increased the COX-2/PGIS expression. Most notably, the release of PGI_2_ was significantly higher in arteries treated with BMEPCs, and inhibition of COX-2, but not COX-1, significantly reduced the synthesis of PGI_2_ and its second messenger cAMP. Immunohistochemical staining analysis demonstrated the increase expression of COX-2 protein in the entire vascular wall, including adventitia, media, and endothelium, suggested that in pulmonary arteries, BMEPCs played a stimulatory role on synthesis of COX-2/PGI_2_.

In blood vessels, PGI_2_ was mainly linked to COX-2 induction, produced predominantly by the endothelial cells, and acted in a paracrine manner, due to its very short half-life. PGI_2_ plays an important role as an endogenous regulator of vascular homeostasis: inhibiting platelet aggregation, VSMC proliferation and migration, stimulating VSMC relaxation, and regulating VSMC differentiation [32]. It is likely that all of the function of PGI_2_ help to maintain the integrity of vasculature. Recently, PGIS expression has been shown to be decreased in the remodeled pulmonary arteries containing plexiform lesions in patients with PAH [33], and patients with PAH have significantly decreased production of PGI_2_, but increased production of TXA_2_ and PGE_2_. A previous study has reported that gene transfer of human PGIS could improve MCT-induced PAH in rats [34]. Moreover, in our study, BMEPCs inhibited production of TXA_2_ and did not alter production of PGE_2_, suggested that PGI_2_ was the main prostanoid released from pulmonary arteries stimulated by BMEPCs. Taken together, our data support the selective activation of COX-2/PGIS by BMEPCs.

In conclusion, BMEPCs therapy was effective at attenuating MCT-induced PAH in a rat model and the vasoprotective effects of BMEPCs on pulmonary arteries might be mediated by COX-2/PGI_2_/cAMP pathway. Therefore, this study may provide a novel mechanism to explain the therapeutic effects of BMEPCs transplantation on PAH.
